# Ultrasound-Mediated DNA Transformation in Thermophilic Gram-Positive Anaerobes

**DOI:** 10.1371/journal.pone.0012582

**Published:** 2010-09-04

**Authors:** Lu Lin, Houhui Song, Yuetong Ji, Zhili He, Yunting Pu, Jizhong Zhou, Jian Xu

**Affiliations:** 1 Qingdao Institute of BioEnergy and Bioprocess Technology, Chinese Academy of Sciences, Qingdao, Shandong, China; 2 Institute For Environmental Genomics, Department of Botany and Microbiology, University of Oklahoma, Norman, Oklahoma, United States of America; New England Biolabs, Inc, United States of America

## Abstract

**Background:**

Thermophilic, Gram-positive, anaerobic bacteria (TGPAs) are generally recalcitrant to chemical and electrotransformation due to their special cell-wall structure and the low intrinsic permeability of plasma membranes.

**Methodology/Principal Findings:**

Here we established for any Gram-positive or thermophiles an ultrasound-based sonoporation as a simple, rapid, and minimally invasive method to genetically transform TGPAs. We showed that by applying a 40 kHz ultrasound frequency over a 20-second exposure, Texas red-conjugated dextran was delivered with 27% efficiency into *Thermoanaerobacter* sp. X514, a TGPA that can utilize both pentose and hexose for ethanol production. Experiments that delivered plasmids showed that host-cell viability and plasmid DNA integrity were not compromised. Via sonoporation, shuttle vectors pHL015 harboring a jellyfish *gfp* gene and pIKM2 encoding a *Clostridium thermocellum* β-1,4-glucanase gene were delivered into X514 with an efficiency of 6×10^2^ transformants/µg of methylated DNA. Delivery into X514 cells was confirmed via detecting the kanamycin-resistance gene for pIKM2, while confirmation of pHL015 was detected by visualization of fluorescence signals of secondary host-cells following a plasmid-rescue experiment. Furthermore, the foreign β-1,4-glucanase gene was functionally expressed in X514, converting the host into a prototypic thermophilic consolidated bioprocessing organism that is not only ethanologenic but cellulolytic.

**Conclusions/Significance:**

In this study, we developed an ultrasound-based sonoporation method in TGPAs. This new DNA-delivery method could significantly improve the throughput in developing genetic systems for TGPAs, many of which are of industrial interest yet remain difficult to manipulate genetically.

## Introduction

There have been relatively few reported successes in the transformation of thermophilic, Gram-positive, anaerobes (TGPAs) [1], [2], [3]. Firstly, some thermophilic bacteria are difficult to transform due to the unique features of their cell envelope, the formation of endospore and the low permeability of plasma membrane [4]. Secondly, most of the reported gene transfer protocols for these organisms were based on electroporation [1], [2], [5], where higher electroporation efficiency was limited to only a few laboratories that used sophisticated customer-built cuvettes and pulse generator [2]. Key drawbacks of electroporation include the need for an ion-free condition and the requirement of contact between electrodes and solution. Moreover, DNA electroporation through the membrane is not a mechanical penetration process, because interactions between pore and DNA are involved [6]. This interaction may slow down DNA penetration through cell wall after pulsing, which might expose the foreign double-stranded DNA to high-temperature and reduce its stability and integrity. Thirdly, the fastidious requirements in cell handling and preparation of competent-cells for strictly anaerobic bacteria further exasperated this situation. Therefore, there is a tremendous need for developing a simple, rapid and effective method for genetically transforming TGPAs.

*Thermoanaerobacter* are a group of gram-positive anaerobes with optimal growth temperature of 60–65°C. They have attracted growing attention over the past few years due to their rare capability utilizing both hexose and pentose at high temperature for ethanol production [7]–[11]. The co-culture of the hexose- and pentose- fermenter *Thermoanaerobacter* and cellulose degraders such as *Clostriditium thermocellum*, both TGPAs, fulfills the key requirements in a Consolidated BioProcessing scheme [12], [13] and therefore has been considered one promising strategy for industrial-scale cellulosic ethanol production. One of the *Thermoanaerobacter* strains, X514, was of particular interest. When partnering with selected *C. thermocellum* strains, the X514 strain yield higher ethanol yields than other closely related *Thermoanaerobacter* strains [8], [9]. Furthermore, *Thermoanaerobacter ethanolicus* fermented pentose and hexose for ethanol in a simultaneous and unbiased manner [14], a feature crucial for cost-efficient cellulosic ethanol production but not widely found in currently available ethanologens such as *Saccharomyces cerevisiae*. Recently the genomic sequence of X514 has been completed (GenBank ID: NC010320) and the central metabolic pathways in X514 have been constructed via metabolic flux analysis [9]. However, the sugar to ethanol conversion rate in *Thermoanaerobacter* (<30%) remains low [7], [9]. Along with ethanol, other fermentation by-products were usually generated, including acetate and lactate, which may significantly reduce ethanol yields [7], [9]. Therefore, it is imperative that the genomic, metabolic and physiological understanding be leveraged towards genetic and genomic engineering of the cells for trait improvement.

Genetic manipulations of TGPAs in general, and of X514 in particular, are often hindered by the lack of a quick and efficient genetic transformation system. A few exceptions reported have involved custom-made electroporation-apparatus that are not accessible to most laboratories [1], [3]. Thus, it is of paramount importance that quick and efficient transformation systems be developed for these organisms. Ultrasound-based sonoporation, an alternative to traditional electroporation for gene transfer, has been extensively studied in eukaryotic transformation and gene therapy [15], [16]. Sonoporation employs acoustic cavitation of microbubbles to create pores on the cell membrane, which enhances delivery of DNA and other macromolecules [17]. When cell membrane is self-repaired, the DNA molecules are thus trapped inside cells. To date, all successful bacterial sonoporation experiments have been carried out only in mesophilic organisms or in Gram-negative organisms such as *Fusobacterium nucleatum, Escherichia coli DH5a, Pseudomonas fluorescens* and *Pseudomonas putida* [17], [18]. Efforts that target thermophilic or Gram-positive organisms have not been successful.

In this study, we established a simple, rapid and effective transformation method for TGPAs based on ultrasound-mediated sonoporation. Transformation was confirmed by detecting the presence or the activities of three introduced heterologous genes that include an antibiotic resistance marker gene, a green fluorescence protein gene and a β-1,4-glucanase gene. The introduced β-1,4-glucanase gene was functionally expressed and conveyed to the host-cell the ability to degrade cellulose, converting it into a prototypic thermophilic Consolidated BioProcessing organism that is not only ethanologenic but also cellulolytic [12]. The advantages of sonoporation-based transformation, particularly the ease and simplicity of procedure, the preservation of cells in indigenous medium and the minimal perturbation to cell viability, suggested this method could be particularly suitable for high throughput development of genetic systems.

## Results

### Effects of ultrasound-exposure duration and electroporation field strength on delivery efficiency of fluorescent dextran

**Figure 1:**
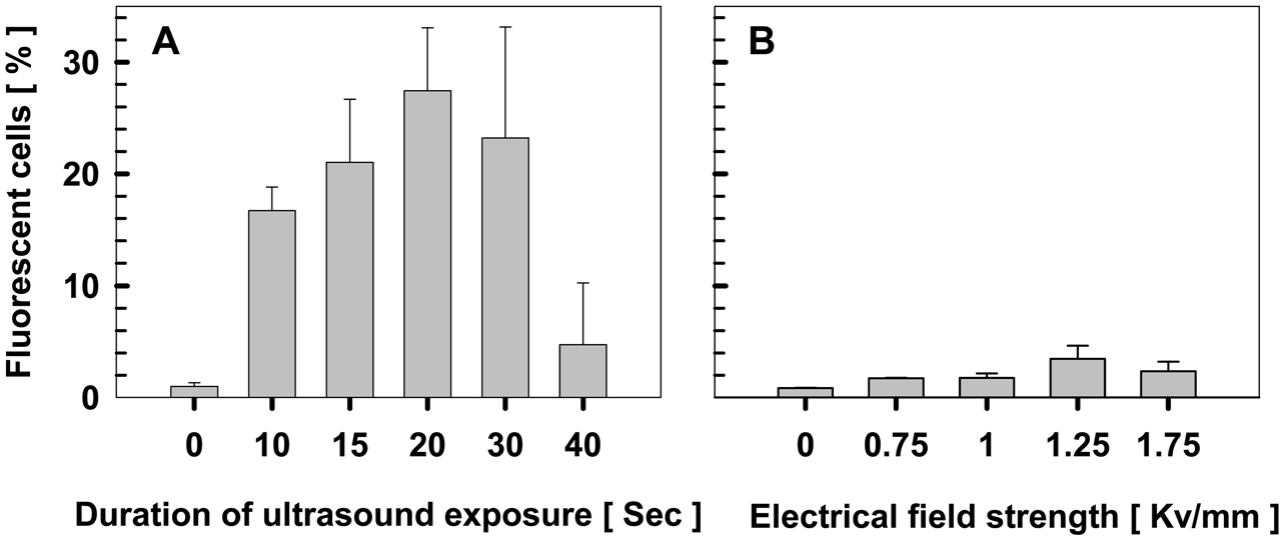
Effects of ultrasound exposure duration or electrical field strength on the delivery efficiency of Texas-red labeled dextran. **A.** Effects of sonoporation-exposure duration on delivery efficiency of Texas-red labeled dextran. **B.** Effects of electrical field strength on delivery efficiency of Texas-red labeled dextran. The ratio of Texas-red-featured, fluorescent X514 cells to total cells were quantified by five randomly selected view-zones under fluorescent microscope. Standard deviations are also shown.

**Figure 2:**
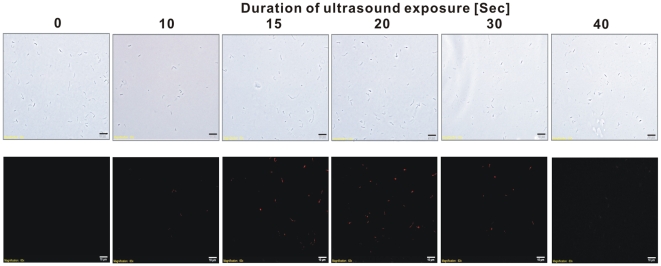
Delivery of Texas Red-conjugated dextran into *Thermoanaerobacter sp.* X514 by sonoporation. The sonoporation experiments were conducted with exposure durations ranged from 0 to 40s. Cells were visualized under Olympus-BX51 microscope with phase lights (upper-row figures) and blue lights (lower-row figures). Scale bar represents 10 µm.

To develop and optimize the sonoporation method for TGPAs, we first tested the delivery of fluorescent Texas-red conjugated dextran into native *Thermoanaerobacter* sp. X514 cells using a Branson B200 Sonifier with fixed ultrasound frequency and power (40 kHz, 19 W). The delivery efficiency of Texas-red was measured as the proportion of fluorescent cells to all cells in a view-zone under the microscope [18]. The results showed that the population of fluorescent cells increased with extended ultrasound exposure, and reached the maximum after 20 seconds of exposure (Fig. 1A; Fig. 2). It is concluded that optimal ultrasound-exposure duration for sonoporation in the Branson B200 sonifier is 20 seconds (Fig. 1A; Fig. 2). Under this condition, intracellular delivery of the dextran was found in 27.5% of the Thermoanaerobacter cells. This finding suggested that the delivery efficiency of DNA might also reach such a high level by optimizing the parameters such as temperature, frequency, intensity and duration of ultrasound exposure.

We next tested the Texas-red delivery using a BioRad Micro Pulser electroporator, in order to compare the transformation efficiencies of sonoporation and electroporation. For the later, additional steps were required, including protoplast generation, iterative centrifugation and multiple rounds of washing under anaerobic conditions, to ensure very low ion concentration and maximal efficiency (S*ee* **Materials and Methods**). Optimal conditions were located by monitoring the delivery of fluorescent dextran into X514 upon pulsing under 0 to 1.75 Kv/mm electrical fields. The result showed that delivery efficiencies increased with elevated field strengths up to 1.25 Kv/mm and reached a maximum efficiency of 3.4% (**Fig. 1B**), which was 8-fold lower than the sonoporation method (**Fig. 1A**).

### Effects of ultrasound and electroporation pulse on cell viability and plasmid integrity

**Figure 3:**
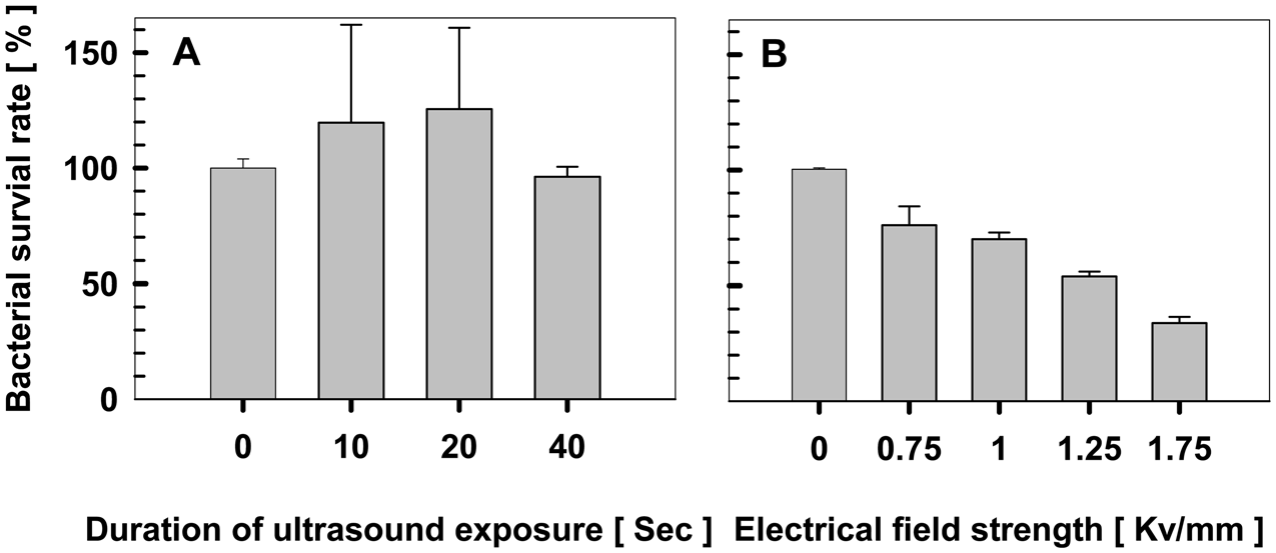
Survival rate of *Thermoanaerobacter sp.* X514 after sonoporation or electroporation. **A.** Survival rate of X514 after sonoporation under exposure durations ranged from 0 to 40s. **B.** Survival rate of X514 after electroporation under field strengths ranged from 0 to 1.75 Kv/mm. Viable colonies were determined by plating cells on agar plates. The percent viability (survival rate) was calculated as described in **Materials and Methods**.

To examine whether ultrasound has deleterious effects on cells, *Thermoanaerobacter* X514 cells were sonified for durations ranging from 0 to 40s and plated on agar plates without antibiotics. Results showed that cell viability was not compromised under the durations of exposure investigated (**Fig. 3A**). Interestingly, exposure durations between 10s and 20s resulted in 20% higher colony counts in cell viability assays, likely due to the dispersal of clumped cells under brief ultrasound treatments [17]. These data suggest that ultrasound can be used to efficiently deliver plasmid DNA into *Thermoanaerobacters* without incurring cell damage.

The post-electroporation survival rate of X514 was also investigated by plating after electroporation under field strengths ranging from 0 to 1.75 Kv/mm. The result showed that the survival rate was drastically reduced under elevated voltages (**Fig. 3B**), indicating that cell membranes might undergo irreversible structural changes and cell damage ensued under high electrical field strength.

Ultrasound, at higher intensity (e.g. 400∼1000 W) or longer exposure, has been widely applied to DNA fragmentation in aqueous solutions as ultrasound can break hydrogen bonds and rapture single- or double-strands in the helix [19]. To test the impact of ultrasound on the plasmid DNA to be delivered, the plasmid pIKM1 was sonified for durations ranging from 0 to 40s. We found that plasmid integrity was not compromised by sonification at the durations of exposure investigated, as determined by pixel densitometry of supercoiled and linearized DNA bands on the gel (**Fig. S1**). Compared with electroporation, the higher cell viability (**Fig. 3A**) under sonification and the apparent preservation of post-sonification plasmid integrity (**Fig. S1**) were consistent with the much higher percentage of Taxas-red-transformed fluorescent cells observed under sonoporation (**Fig. 1A**). These results showed that sonoporation could be superior to electroporation in transformation efficiency and cell viability preservation.

### Methylation modification assay

The state of foreign DNA to be delivered also determines transformation efficiency. DNA methylation is a chemical modification system defending against foreign-DNA invasion. It was widely found in higher organisms, but varies in frequency in thermophilic bacteria. For instance, foreign DNA must be methylated before it can be introduced into *C. thermocellum* [2] via electroporation, while foreign-DNA methylation was not required for electro-transforming *Thermoanaerobacterium saccharolyticum* JW/SL-YS485 [1].

**Figure 4:**
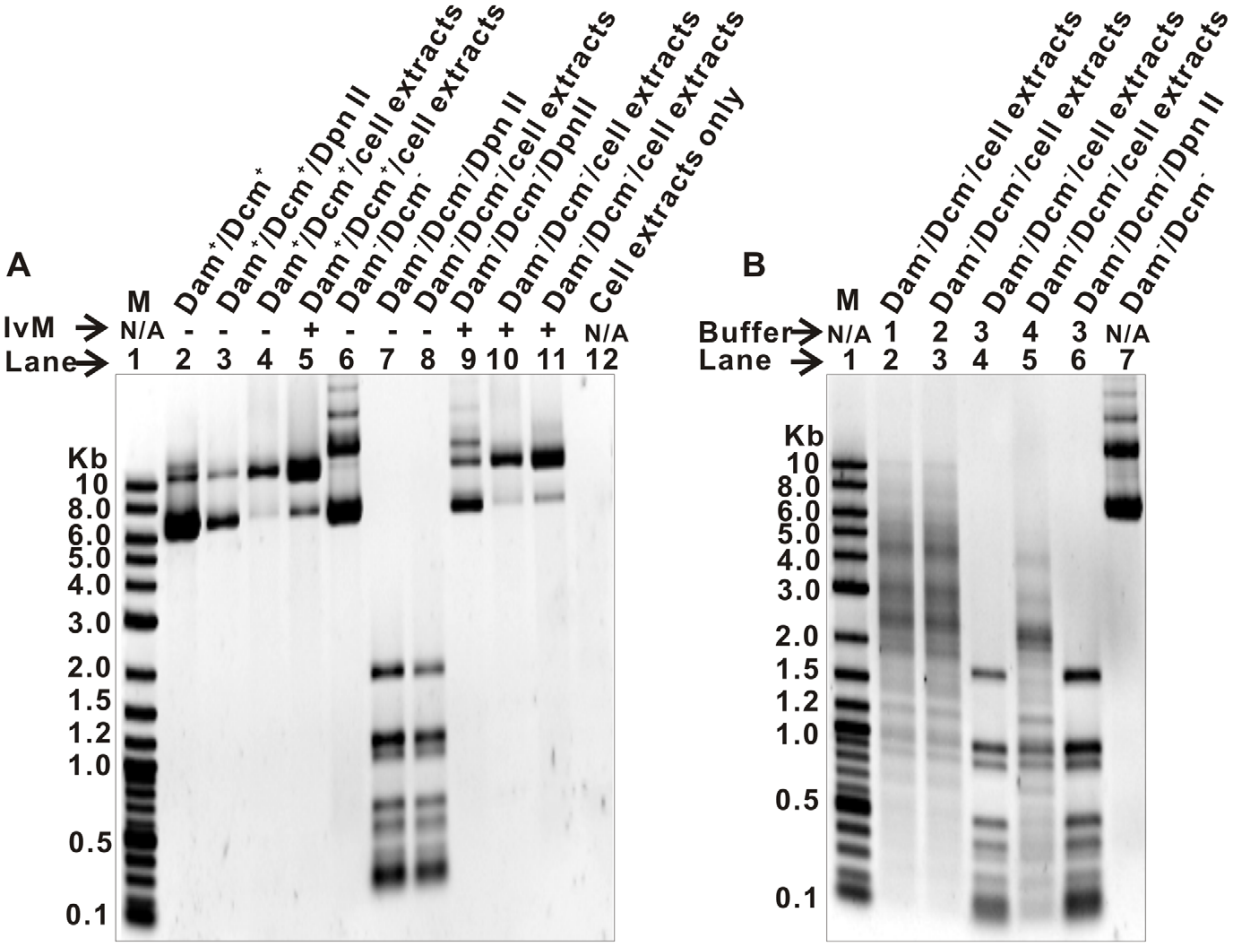
Agarose gel electrophoresis of pHL015 plasmids employed in methylation assay. **A.** The pHL015 plasmids (Lanes 2 to 11) were extracted from either Dam^+^/Dcm^+^ *E. coli* DH5α or Dam^−^/Dcm^−^ *E. coli* JM110. The plasmids were separated on 1% agarose gel after incubation with X514 cell extracts (Lanes 4, 5, 8, 10 and 11) or DpnII (Lanes 3, 7 and 9). *In vitro* methylated (IvM) plasmids are marked with “+” on top of this figure (Lanes 5 and 11: methylated by X514 cell extracts; Lanes 9 and 10: methylated by a commercial Dam methyltransferase). **B.** The pHL015 plasmids (Lane 2 to 7) were extracted from Dam^−^/Dcm^−^ *E. coli* JM110. The plasmids were separated on 1% agarose gel after incubation with X514 cell extracts in the NEB restriction enzyme buffer 1, 2, 3 and 4 in parallel (Lane 2 to 5). The NEB buffers used are illustrated on top of this figure. M: molecular weight marker. N/A: not applicable.

To determine whether enzymes from *Thermoanaerobacter* sp. X514 could degrade foreign plasmid DNA, cell extracts of X514 were incubated with various forms of pHL015 (**Fig. S2B**). Methylation-competent *E. coli* DH5α (Dam^+^/Dcm^+^), but not the methylation-deficiency mutant *E. coli* JM110 (Dam^−^/Dcm^−^) cells, methylated pHL015 *in vivo*. The methylated pHL015 extracted from *E. coli* DH5α was not degraded by X514 cell extracts while the unmethylated pHL015 extracted from *E. coli* JM110 was readily cleaved by X514 cell extracts (**Fig. 4A**, Lane 4 and Lane 8 respectively). *In vitro* methylated pHL015, extracted from either species of *E. coli,* resulted in no degradation by X514 cell extracts (**Fig. 4A**, Lane 5, Lane 10 and Lane 11 respectively). These findings suggest that methylation of DNA is required for protection against degradation in X514 cells. To examine whether the enzyme activity underlying plasmid degradation in X514 is buffer dependent, the degradation of Dam^−^/Dcm^−^ pHL015 was evaluated in the presence of the New England Biolabs (NEB) restriction enzyme Buffer 1, 2 3 and 4. Nonspecific degradation of pHL015 was observed in the presence of NEB Buffer 1, 2 and 4 (**Fig. 4B** **, Lane 2, 3 and 5**), suggesting buffer dependency of the enzyme activity.

To identify the putative restriction methylation (RM) system(s) in X514, we took advantage of the complete genome sequence of *Thermoanaerobacter* X514 (Genbank ID: NC010320). From the genome, a total of three putative genes encoding DNA methyltransferases (Teth514_0458/1737/1828) were identified, which, based on our functional annotations, all methylate adenines at their N6 positions in GATC sequences [20]. We hypothesized that the GATC sequences are one signal for cleavage recognized by the X514 RM systems. To test this hypothesis, we compared the gel electrophoresis patterns of pHL015 (methylated or unmethylated) resulted from two treatments, one by X514 cellular extracts and the other by DpnII, a commercially available enzyme that specifically cleaves GATC when the A is not methylated. While X514 extracts did not degrade *in vivo* or *in vitro* methylated pHL015, DpnII similarly did not degrade methylated pHL015 from *E. coli* DH5α (**Fig. 4A**, Lane 3). However, treatments by X514 extracts or DpnII yield identical restriction profiles for unmethylated pHL015 from *E. coli* JM110 (**Fig. 4A**, Lane 7 and Lane 8 respectively). The results suggest that X514 cells harbor at least one restriction enzyme that digests unmethylated GATC sequences. This finding was consistent with the predicted function of the three N6 adenine-specific DNA methyltransferases found in X514 genome. A DpnII-like isoschizomer was previously predicted in REBASE of NEB (http://rebase.neb.com/rebase/rebase.html) [21] with an official name of TspX514ORF458P and its companion methylase M.TspX514ORF458P (The letter “P” indicates that the function is still putative). Taken together, it is concluded that the adenine methylation modification system in *Thermoanaerobacter* sp. X514 was active and is likely the major restriction modification system in this organism, suggesting that methylation of foreign DNA by specific methylases or cell extracts [22] is essential prior to introducing into X514 cells.

### Transformation and heterologous expression of green florescence protein (GFP) gene and β-1.4-endoglucanase gene in Thermoanaerobacter sp. X514 by sonoporation

**Figure 5:**
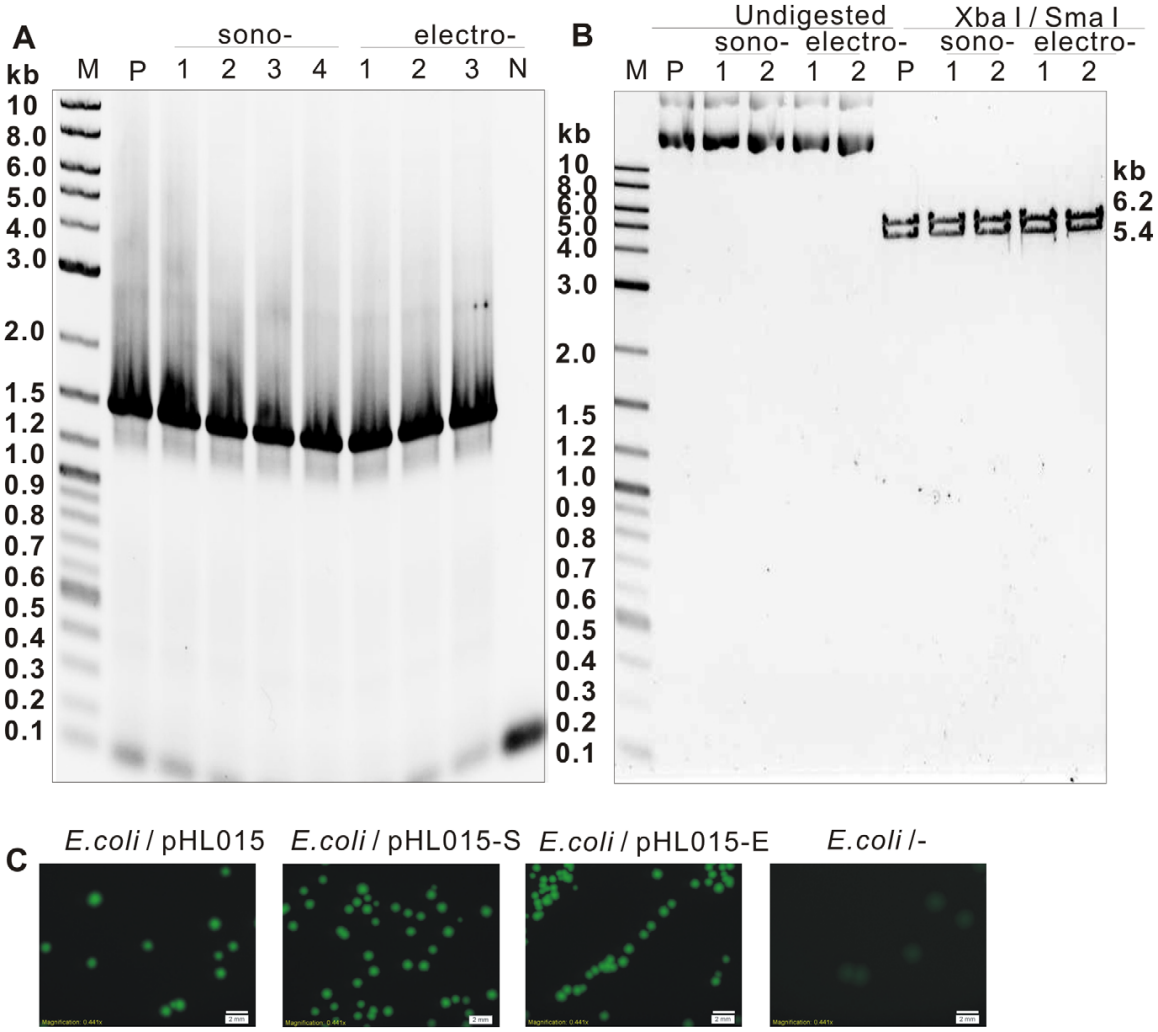
Confirmation of transformation of *Thermoanaerobacter* cells using PCR analysis and plasmid rescue experiments. **A.** PCR amplication of the kanamycin resistance marker-gene from pIKM2. The PCR products were amplified from DNA preparations of pIKM2-transformed X514 cells by sonoporation (sono-) or electroporation (electro-). The plasmid pIKM2 and wild-type X514 DNA were used as PCR template for positive (P) and negative (N) controls, respectively. **B.** Plasmid-pIKM2 rescue experiments. The plasmid DNA were extracted from X514-pIKM2 transformants, and “rescued” in *E. coli* (see **Methods**). The *E. coli*-rescued plasmids were digested by XbaI /SmaI and separated on 1% agarose gel. Plasmid pIKM2 was used as positive control (P). **C.** Plasmid-pHL015 rescue experiments. The plasmid DNA were extracted from X514-pHL015 transformants, and rescued in *E. coli*. The *E. coli* cells harboring rescued plasmids were visualized under Olympus-BX51 microscope equipped with a CCD. Scale bar represents 2 mm. *E. coli*/pHL015-S: the *E.coli*-rescued plasmid that was transformed into X514 by sonoporation. *E. coli*/pHL015-E: the *E.coli*-rescued plasmid that was transformed into X514 by electroporation. “M”: molecular weight marker.

To test the efficacy of sonoporation in delivering heterologous double-stranded plasmids into Thermoanaerobacter, methylated pIKM2 (which encodes a clostridial β-1.4-endoglucanase gene, **Fig S2A**) or pHL015 (which encodes a jellyfish *Aequorea Victoria gfp* gene, **Fig. S2B**) were delivered into *Thermoanaerobacter* sp. X514 by sonoporation, and by electroporation in parallel. Efficiencies of sonoporation and electroporation were 6×10^2^ and 1×10^2^ CFU/µg plasmid DNA, respectively, based on the experimentally determined optimal conditions described above. Multiple experiments to confirm the transformation were carried out. The kanamycin resistance gene on pIKM2 was detected by PCR from the X514 colonies transformed via either sonoporation or electroporation (**Fig. 5A**). However, the pIKM2 DNA extracted from X514 transformants was not visible on EtBr-stained agarose gels (data not shown), likely due to low plasmid copy numbers in the host cell or inefficient recovery of pIKM2 from the Gram-positive X514 during plasmid DNA extraction. To address this ambiguity, “plasmid rescue” experiments was carried out. The plasmid pIKM2 extract from the Km^r^ X514 colonies was transformed into *E. coli* cells, followed by propagation and extraction from the *E. coli*. The XbaI/SmaI restriction digestion profile of the pIKM2 originally delivered into X514 was identical to that of the pIKM2 recovered from *E. coli* transformants (**Fig. 5B**), demonstrating that the pIKM2 derived from X514 was intact.

However, the signature fluorescence of GFP was not observed in X514 under fluorescent microscope (data not shown), suggesting that the *gfp* gene encoded on pHL015 was either not expressed or was not functional in the hosting X514 cells. Causes for the later possibility might include protein structural-changes under high temperature or in the absence of molecular oxygen, both features of the *Thermonanaerobacter* growth [23]. To test this possibility, a plasmid rescue experiment was performed and the plasmid DNA extracts from the pHL015-transformed X514 were subsequently transformed into *E. coli*. GFP fluorescence was visualized in the *E. coli* transformants. This result suggests that albeit GFP protein is not a suitable marker gene for thermoanaerobes, the transformation of X514 might have been successful (**Fig.** **5C**).

**Figure 6:**
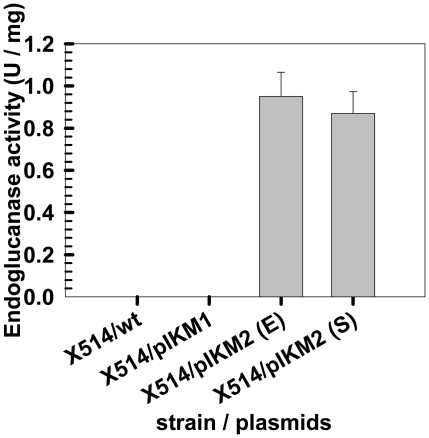
Heterologous endoglucanase activity of *Thermoanaerobter sp.* X514 transfomants. The endoglucanse activity was determined as described in **Materials and Methods**. The X514 was transformed by electroporation (E) or sonoporation (S) respectively. Wild-type X514 and pIKM1 transformants were used as negative controls. All experiments were performed in triplicate, with standard deviations shown.

However, the possibility that plasmids were surface-attached with, but not ‘into’, the X514 cell could not be excluded. In fact, excess amounts of plasmid DNA (5 µg) were used for each X514 transformation and spread together with cells on agarose plates. To rule out this possibility, we sought direct evidence for successful transformation by expression of a heterologous (clostridial) β-1.4-endoglucanase gene in X514 and detection of the expressed endoglucanase activity in the presence of cellulose (CMC-Na). Result showed that X514 transformed with plasmid pIKM2 by sonoporation or by electroporation exhibited endoglucanase activities of 0.87 U/mg and 0.95 U/mg respectively, whereas the X514 wild type and X514 transformed with the pIKM1 plasmid (one without the clostridial β-1.4-endoglucanase gene) could not degrade cellulose (**Fig. 6**). These results demonstrated that the pIKM2 encoding β-1.4-endoglucanase was indeed delivered via sonoporation into and functionally expressed in *Thermoanaerobacter* sp. X514.

## Discussion

The most commonly used methods of DNA transformation in bacteria are conjugation and electroporation. Typically, a specific DNA donor strain is required to achieve DNA transfer in conjugation. However, the lack of suitable donor strains makes conjugation difficult for thermophilic low G/C organisms such as *Thermoanaerobacter*. Thus most of the described gene transfer protocols for these types of organisms are based on electroporation [1], [2], [5], [24]. However, success with these methods was limited to only a few laboratories, which used custom-built cuvettes and pulse generator [2], [3] that are not accessible to most laboratories. In this study, we developed an efficient and quick transformation system for Gram-positive thermophiles via ultrasound-based sonoporation. The method was successfully employed in *Thermoanaerobacter* X514 with a transformation efficiency of 6×10^2^ CFU/µg plasmid DNA. The efficiency is much higher than that from a parallelly performed, yet much slower and more tedious, electroporation method (1×10^2^ CFU/ug plasmid).

### The advantages of ultrasound-based sonoporation

There are several advantages of sonoporation-based over electroporation-based transformation. Firstly, the procedure and protocol for the former is much less cumbersome and easer to follow (*See* **Materials and Methods**). In contrast, the later necessitates adding glycine to the medium to induce spheroplast formation [5], or by using cell wall-weakening agents such as isonicotinic acid hydrazide [1], [2]; moreover, laborious pretreatment steps involving chilling during cell collection and repetitious washing under anaerobic conditions are required. Thus sonoporation is particularly advantageous for handling strictly anaerobic bacteria, as the likelihood of exposing the cells to aerobic environments is minimized. Secondly, ultrasound sonoporation is a non-cell-contacting and less invasive method that is thus friendly to remote-control. The acoustic cavitation of microbubbles can be directly applied to indigenous cells in their native growth media, where the microbubbles permeate cell membranes and facilitate the delivery of DNA and other macromolecules [17]. Cellular manipulation can be achieved outside of an anaerobic glove box simply by immersing a sealed glass vial into a sonifier chamber, without any cell transfers or direct contact of the cells with anything other than the indigenous growth media. In contrast, in electroporation, cells must be removed from their native growth medium, exposed to low-ionic strength buffer, transferred to and held in a specialized electro-pulse cuvette for electro-pulsing; moreover, the whole process has to take place in a specialized anaerobic glove box. Thirdly, supported by our study, ultrasound treatment does not reduce viability as cells can quickly repair membranes, whereas cell survival is drastically reduced after electroporation due to irreversible membrane damage [25]. Fourthly, sonoporation eliminates the need for expensive or specialized instruments and facilities. The only essential equipment is a commercially available and inexpensive ultrasound bath, in this study a Branson B200 (40 kHz, 19W). While in electroporation of obligate Gram-positive anaerobes such as some thermophilic *Clostridia*, custom-made electroporators that are until now not accessible to most laboratories are required [1], [3], and a large anaerobic workstation for competent cells preparation and applying electro-pulses has to be equipped.

These advantageous features of sonoporation-based transformation suggested that this method can be readily adapted for high-throughput or remotely controlled screening and testing of genetic transformation schemes, where a large number of transformation experiments are performed in parallel, with or without robots. Furthermore, the ultrasonic DNA transfer is highly scalable and can be applied to large bioreactors and natural environments. This key feature could find potential applications for outdoors, large-scale, DNA-transformation-based modulation of microbial-community structure and function.

### Additional barriers for DNA transformation towards TGPAs

The unique physiological features of TGPAs represent additional barriers for DNA transformation. Firstly, the restriction-methylation systems (RM) of bacterial cells may digest improperly methlyated incoming DNA [26], [27], which renders low transformation efficiency in recalcitrant hosts. As increased transformation efficiency using unmethylated DNA is rare [28], the foreign genetic materials should be methylated prior to delivery to prevent cleavage by the RM systems. Without a complete genome, identification of the specific methylase for DNA modification to resist restriction enzymes in the host cells is typically a tedious and cumbersome trial-and-error exercise. Here, we demonstrated a whole-genome-sequence-based strategy to quickly identify and confirm the particular restriction systems. A DpnII-like restriction system which digests unmethylated 5′-GATC-3′ sequence was identified in *Thermoanaerobacter* sp. X514. Together with the previously reported MboI-like RM systems in *C. thermocellum* [2], [29], our finding suggested such RM systems might be one common barrier to transformation in TGPAs. The exonuclease activities in thermophiles are species/strain, temperature and buffer dependent. For instance, in contrast to *Clostridium thermosaccharolyticum* ATCC 31960, little nonspecific exonuclease activity was observed in *C. thermocellum* ATCC27405 (clear bands on agarose gel; [29]). Even for *C. thermocellum* ATCC27405, nonspecific degradation of linear DNA was observed at 63°C but not at 55°C or 60°C [29]. In our study, we observed nonspecific degradation of unmethylated plasmid by X514 cell extracts in the presence of NEB Buffer 1, 2 and 4, but not 3 (**Fig. 4B**). Secondly, the replication origin of transformed plasmid DNA is another important factor influencing plasmid stability inside the hosting thermophilic cells. To date, no native plasmid replication origin has been identified in thermophiles. The exogenous plasmid replicon used in *Thermoanaerobacter* and *C. thermocellum* was isolated from the mesophilic *Bacillus subtilis* plasmid pIM13 [1], which might result in low copy-number of the plasmids in thermophiles. The low copy-number of pIKM1, which was derived from pIM13, could underlie our inability to visualize the isolated plasmid from transformed Thermoanaerobacter on agrose gel. This result was consistent with the observations in *C. thermocellum* [2]. However, the isolated pIKM1 could be gel-visualized from the transformed Thermoanaerobacterium [1]; while plasmid pTE16, constructed not from *Bacillus subtilis* but from the replicon origin of *Clostridium perfringens* plasmid pIP404, could be abundantly isolated from transformed Thermoanaerobacter [5]. Therefore, the copy number is dependent on the specific strains and plasmids used. Thirdly, the paucity of thermostable antibiotics and their corresponding resistance marker-genes have limited the genetic manipulation of thermophiles. Without such thermostable genetic marker systems, false positive colonies would grow on plates after transformation (data not shown). Thus, in this study we attempted a fluorescence-based reporter system by constructing pHL015 which harbors a *gfp* reporter gene. However, GFP was not functional in the thermoanaerobic X514 due to the high temperature or the lack of molecular oxygen [23]. Another plasmid encoding the fluorescent marker *PpFbFP*, which was reported functional at both aerobic and anaerobic conditions [30], quickly quenched at temperatures above 40°C (data not shown). Eventually, we developed the reporter system consisting of clostridial β-1,4-glucanase gene whose expression was driven by a *Thermoanaerobacter*-compatible promoter. With this strategy, not only transformation but also heterologous expression were directly and conclusively validated via measuring the enzymatic activity in the transformed host cells. It is note-worthy though, that as detection and measurement of enzyme activities is typically indirect and much more cumbersome than those of *in situ* fluorescent signals, identification and development of fluorescent-protein marker-genes that are functional in thermophilic anaerobes is warranted.

### Application of sonoporation

The sonoporation method and reporter systems developed and validated in this study can be used as a generalized strategy to over-express foreign or native genes in thermophilic anaerobes, for example, by replenishing the carbon flow to ethanol fermentation pathway [9]. Apart from that, they might also be a useful for creating gene deletions involved in branched fermentation pathways. Traditional methods use suicide vectors to achieve in-frame deletions via homologous recombination. However, this requires much higher transformation efficiencies, usually above ∼10^5^ CFU/µg DNA [31]. Although the transformation efficiency of sonoporation is comparatively low, sonoporation has been successfully used to construct allelic-exchange mutant in the Gram-negative bacterium *Fusobacterium nucleatum* [18]. Furthermore, there are gene-knock-out methods that might exploit the characteristics of sonoporation-based transformation. For example, the recently developed ClosTron, a gene-knock-out system alternative to homologous recombination, is based on Group II introns and does not require a high transformation efficiency [32]–[35]. To date, however, this knock-out system has merely been used in mesophilic organisms [33], [34], [35]. Sonoporation-based transformation and ClosTron, when coupled together, might be a promising strategy for gene-knockouts in thermophiles. Further optimization of this protocol to increase transformation efficiencies in thermophiles or Gram-positive bacteria may include optimizing sonoporation parameters (e.g, acoustic pressure amplitude, sonoporation frequency and intensity) or addition of calcium and Definity to control pore size of cell membrane [36], [37], [38].

In conclusion, this study marks the development of a sonoporation-based transformation method for TGPAs. Our work expands the presently limited repertoire of genetic tools for modifying and engineering TGPAs. Furthermore, the advantages of sonoporation suggested it could be readily adapted and particularly suitable for applications where *in situ* cell-transformation, high throughput or remote control is preferred or even required. In the present days, for the microbial world in general and for thermophilic Gram-positive bacteria in particular, the much fewer number of currently available genetics systems contrasts sharply with the rapid and readily availability of sequenced genomes. Sonoporation-based transformation hold the promise to significantly elevate the throughput of genetic system development and contribute to bridging the enormous gap between genome sequences and manipulation of the genomes.

## Materials and Methods

### Bacterial strains and growth conditions

*Thermoanaerobacter* sp. X514 was cultivated in reinforced clostridial medium (RCM, Oxoid) anaerobically at 60°C. For growth on plates, 0.7% Gelrite Gellan Gum (Sigma) was added to RCM medium. *Escherichia coli* strains were grown in Luria-Bertani (LB) medium. When required, the medium was supplemented with kanamycin, 250 µg/ml for *Thermoanaerobacter* sp. X514 and 50 µg/ml for *E. coli*.

### Delivery of fluorescent dextran into Thermoanaerobacter sp. X514 by sonoporation

*Thermoanaerobacter* sp. X514 cells were harvested at mid-log phase, washed and resuspended in phosphate-buffered saline (PBS, pH 7.0) buffer supplemented with 0.1 mM MgCl_2_ and 0.1 mM CaCl_2_ [18]. The cell suspension at 10^9^ cells/ml was then mixed with Texas Red-conjugated dextran (70 kDa; Invitrogen) to a final concentration of 1.25 mg/ml in a flat-bottom glass vial. The vials were immersed in the water bath at the center of the ultrasound device (Branson B200, 40 kHz, 19W) and exposed to ultrasonic waves for durations ranging from 0 to 40 seconds at room temperature. Cells were washed for four times in 500 µl PBS buffer, collected by centrifugation at 4,000 g ×10 min and then were examined under an Olympus-BX51 fluorescence microscope. Viable counts were determined as the number of colony forming units (CFU) after plating diluted cells on RCM-Gelrite agar plates. Percent viability was calculated as viable counts divided by counts in a control, i.e., without sonoporation exposure.

### Delivery of fluorescent dextran into Thermoanaerobacter sp. X514 by electroporation

For the preparation of electro-competent host cells, *Thermoanaerobacter* sp. X514 was grown in 100 ml N_2_-flushed medium. Glycine and sucrose were added into the early log-phase culture with final concentrations of 0.053 M and 0.27 M respectively. The culture was propagated at 60°C until the spheroplast morphology could be visualized under a light microscopy (**Fig. S3**). Cells were harvested by centrifugation at 3,500 g for 10 min at 4°C, washed three times in an anaerobic chamber using chilled electroporation buffer (0.27 M sucrose and 10% glycerol). Such electro-competent cells were resuspended in 2 ml of the same buffer. Texas Red-conjugated dextran was added to the cell suspension for a final concentration of 1.25 mg/ml. Approximately 100 µl of the mixture was transferred to a cuvette (Bio-Rad, 2 mm) and electro-pulsed with 10 µF, 600 Ω and voltages ranging from 0.75 Kv/mm to 1.75 Kv/mm, using a Bio-Rad Micro-Pulser. The cells were then washed four times in electroporation buffer and examined under a fluorescence microscope, as described above.

### Examination of restriction endonuclease activity in host cells

Cell extracts of *Thermoanaerobacter* sp. X514 were prepared from stationary-phase cells as described previously with minor modifications [22]. Briefly, cells from 100 ml stationary-phase culture of X514 were collected by centrifugation (3,500 g for 10 min), washed once with 100 ml chilled PENP buffer (10 mM KH_2_PO_4_-K_2_HPO_4_, 10 mM EDTA, 50 mM NaCl and 0.2 mM PMSF, pH 7.0), and then resuspended in 4 ml of the same buffer. The cells were chilled and maintained in an ice bath during cell-structure disruption in a Branson 450 sonifier (400W). Cellular debris was separated by centrifugation (10,000 g for 15 min) and the extract (supernatant) collected. Aliquots of 3 ml extract were mixed with 3 ml 100% glycerol and 0.6 ml of BSA (1 mg/ml) and then stored at −20°C before analysis. The DNA digestion assay was performed by mixing 0.5–1.6 µg of plasmid DNA, 4 µl cell extract and appropriate restriction endonuclease buffer to a final volume of 25 µl, and then incubated at 60°C for 4 h.

### *In vitro* methylation of DNA

The DNA modification assay was performed as described elsewhere [22] by combining 53 µl TNE buffer (50 mM Tris pH 7.5, 50 mM NaCl, 10 mM EDTA), 10 µl SAM (0.8 mM), 1 µl BSA (10 mg/ml), 25 µl X514 cell extract and 3 µg plasmid DNA (pHL015) prepared from *E. coli* DH5α (Dam^+^/Dcm^+^) or JM110 (Dam^−^/Dcm^−^) in a final volume of 100 µl. The mixture was incubated at 60°C for 16 h. As a control for methylation experiments, a commercial Dam methyltransferase was purchased from New England Biolabs. When the commericial Dam methyltransferase was used, the reaction condition was 37°C for 4 h (based on the enzyme product manual). Methylated DNA fragments were purified by the QIAquick PCR purification kit (Qiagen).

### Construction of plasmid pIKM2 and pHL015

*E. coli*-*Thermoanaerobacter* shuttle plasmid pIKM1 was kindly provided by Dr. Juergen Wiegel (University of Georgia, Athens). A 5.4 kb PCR fragment encoding the 4.5 kb endo-β-1, 4-glucanase gene flanked by its 651 bp promoter and 142 bp transcription terminator region was amplified from the genome of *Clostridium thermocellum* ATCC27405 by a primer-pair (fwd: 5′- CCGTCTAGAATTTTTGCGTATGGATTGTG-3′ and rev: 5′- GGTCCCGGGCTGTTTCCCGTTTTTGTTTT-3′) and subsequently ligated into pIKM1 through XbaI and SwaI. The resulting plasmid was named pIKM2 (**Fig. S2A**). The plasmid pHL015 (**Fig. S2B**) was constructed by inserting the NcoI/NsiI flanked fragment of pIKM1 into similarly digested plasmid pML523, which was a gift from Dr. Michael Niederweis (University of Alabama at Birmingham).

### Plasmid DNA integrity assay

To examine whether sonoporation compromised the integrity of the to-be-delivered foreign DNA, approximately 5 µg of pIKM1 DNA were added to 500 µl PBS buffer in a flat-bottom glass vial. The vials were exposed to 40 KHz ultrasound for durations from 0s to 40s. After sonoporation, DNA samples were transferred to 1.5 ml Eppendorf tubes containing 50 µl 3M NaAC and 1 ml 100% ethanol. Samples were incubated at −20°C for 2 h and then centrifuged at 12,000 g for 20 min. Supernatants were discarded and DNA pellets were dried in air. The DNA pellets were then dissolved in 20 µl nuclease-free water and separated on a 1% agarose gel.

### Delivery of methylated pIKM2 and pHL015 plasmids into Thermoanaerobacter sp. X514 by sonoporation and electroporation

For sonoporation-based transformation, 500 µl cell suspensions at approximately 1×10^9^ CFU/ml were mixed with *in vitro* methylated pIKM2 or pHL015 (5 µg) plasmids in a flat-bottom glass vial. The vial was placed at the center of the ultrasound device and exposed to 40 kHz ultrasound for 20 seconds. The mixture was then transferred to a Hungate tube containing 5 ml fresh RCM medium. Post-sonoporation cells were allowed to recover at 45°C for 5 h and then were diluted, mixed with melted warm RCM-agar medium supplemented with 250 µg/ml kanamycin and poured onto Petri dishes inside an anaerobic glove box (Coy Lab). The agar plates were incubated for 5–7 days in an anaerobic jar at 45°C. The temperature is not raised to the optimal growth temperature of X514 (60°C), in order to preserve the activity of kanamycin.

For electroporation-based transformation, methylated pIKM2 or pHL015 DNA (5 µg) was mixed with 100 µl X514 competent cells as describe above, added into a cuvette (Bio-Rad, 2 mm) and pulsed in a Bio-Rad micropulser (1.25 Kv/mm, 10 µF and 600 Ω). The cells were then immediately transferred into Hungate tubes with 5 ml fresh RCM medium, incubated at 45°C for 5 h, diluted and mixed with melted warm RCM-agar medium supplemented with 250 µg/ml kanamycin, and then plated onto Petri dishes inside an anaerobic glove box. The agar plates were incubated for 5–7 days in an anaerobic jar at 45°C.

After 5–7 days, single colonies were picked, transferred into RCM-kanamycin liquid medium and incubated at 45°C for 3 days. Plasmid DNA was extracted from *Thermoanaerobacter* sp. X514 transformants using a Qiagen plasmid mini-preparation kit. PCR was performed to detect the kanamycin marker gene in pIKM2 transformants using the extracted DNA as template and the primers km-fwd (5′-CGATAAACCCAGCGAACCAT-3′) and km-rev (5′-CAAATTCCTCGTAGGCGCTC-3′) with an expected PCR product size of 1470 bp. In addition, the presence of pIKM2 in Km^r^ transformants of X514 was verified by “DNA rescue” experiments. In these experiments, all extra-chromosomal elements were isolated from X514 and then introduced into and propagated in *E. coli* DH5α; all plasmid DNA was isolated from the *E. coli* using the Qiagen plasmid mini kit, digested by XbaI/SmaI, and separated on 1% agarose gels.

### Enzyme activity assay of the introduced heterologous endo-β-1, 4-glucanase gene

The β-endoglucanase activity was measured as the amount of reduced sugars released from sodium carboxymethyl cellulose (CMC-Na) in the presence of supernatant of the X514/pIKM2 cell-culture filtrate, as the enzyme was secreted. Concentrations of the released sugar were determined by the dinitrosalicylic acid (DNS) method using glucose as the standard, as previously described [39]. One enzyme unit was defined as the amount of protein required to release 1 µmol reduced sugar per minute from the CMC at 50°C. Specific activities are given in units per mg of protein. Protein concentrations were measured by the Bradford assay [40].

## Supporting Information

Figure S1 Impact of ultrasound exposure times on plasmid integrity. The plasmid pIKM1 was examined on 1% agarose gel via electrophoresis, after ultrasound exposure with durations ranged from 0 to 40s in a Branson B200 sonifier. The optimal duration of exposure investigated in this study is indicated with arrow.
(0.46 MB TIF)

Figure S2 Physical maps of plasmid pIKM2 and pHL015. A. pIKM2. The plasmid pIKM2 was constructed by inserting the beta-1, 4-endoglucanase gene into Xba I/SmaI sites of pIKM1 (1) as described in text. B. pHL015. The plasmid pHL015 was constructed by inserting the NcoI/NsiI flanked fragment of pIKM1 into a similarly digested plasmid pML523. The pHL015 harbors a kanamycin resistance gene (aph, from pIKM1), a Bacillus subtilis replicon (pIM-ori, from pIKM1), a hygromycin resistance gene (hyg), a green fluorescence protein gene (gfp), a catechol 2,3-dioxygenase gene (xylE), a levansucrase gene (sacB) and an E. coli replicon (pUC-ori).
(0.24 MB TIF)

Figure S3 Morphology of Thermoanaerobacter sp. X514 cells. A: X514 cells at early growth phase. B: Spheroplast formation of X514 cells in the presence of glycine and sucrose. Photographs were taken using Olympus-BX51 microscope equipped with a CCD. Scale bar represents 10 µm.
(1.13 MB TIF)
