# Supplementary figures and images for: Ultrasound-Mediated DNA Transformation in Thermophilic Gram-Positive Anaerobes
Source: PLoS One. 2010 Sep 4;5(9):e12582. doi: 10.1371/journal.pone.0012582 (PMC2933238; doi:10.1371/journal.pone.0012582)

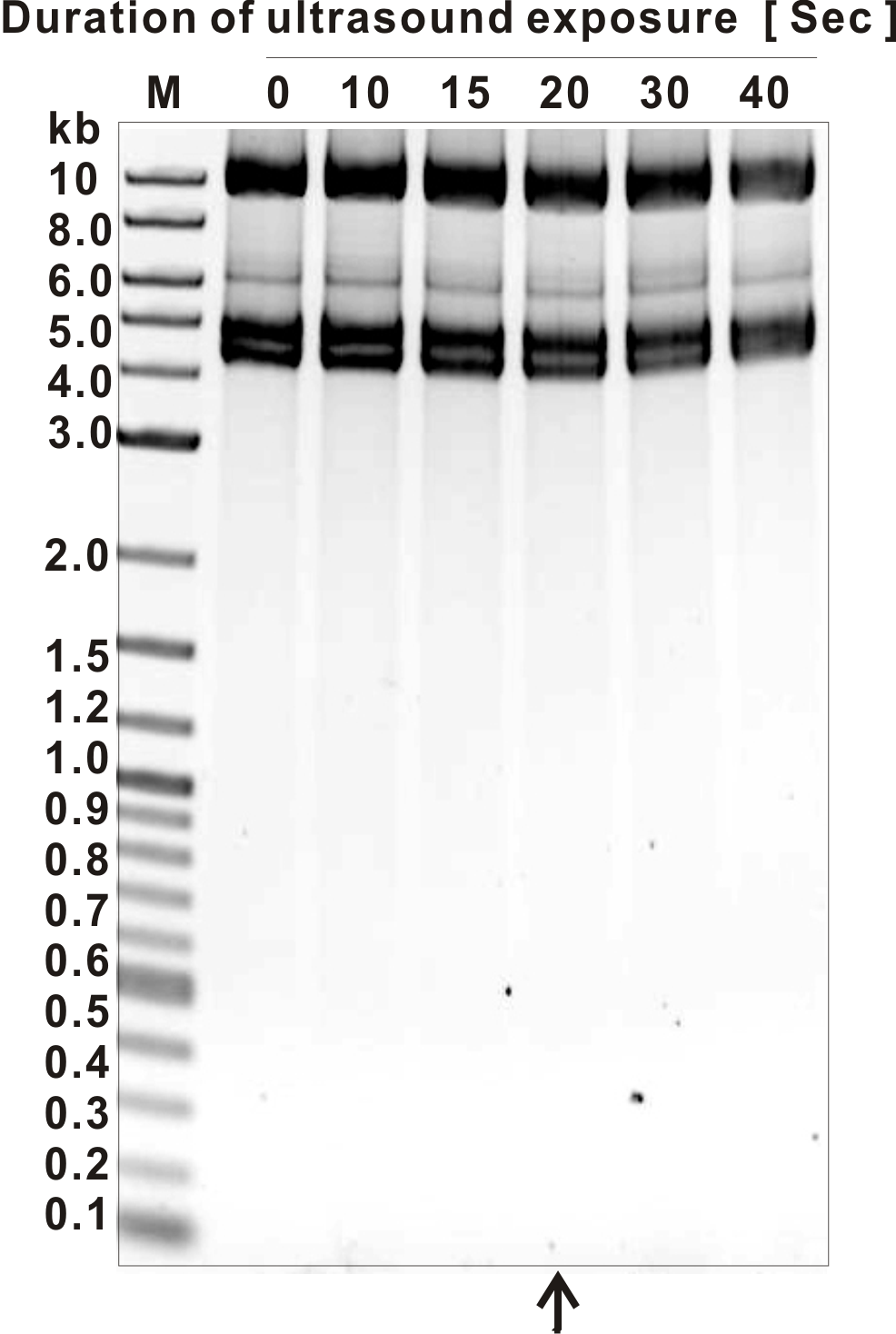

Supplement: Figure S1 — Impact of ultrasound exposure times on plasmid integrity. The plasmid pIKM1 was examined on 1% agarose gel via electrophoresis, after ultrasound exposure with durations ranged from 0 to 40s in a Branson B200 sonifier. The optimal duration of exposure investigated in this study is indicated with arrow. (0.46 MB TIF) [file pone.0012582.s001.tif]

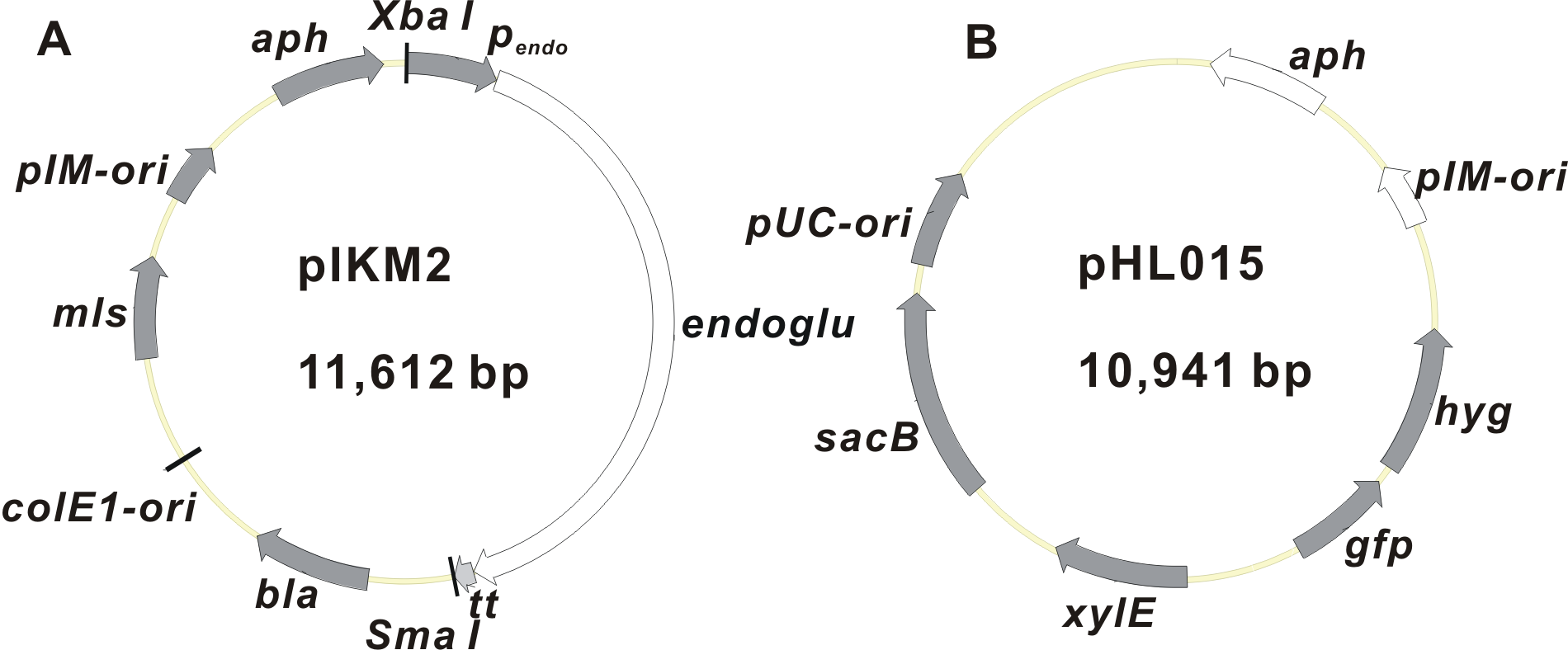

Supplement: Figure S2 — Physical maps of plasmid pIKM2 and pHL015. A. pIKM2. The plasmid pIKM2 was constructed by inserting the beta-1, 4-endoglucanase gene into Xba I/SmaI sites of pIKM1 (1) as described in text. B. pHL015. The plasmid pHL015 was constructed by inserting the NcoI/NsiI flanked fragment of pIKM1 into a similarly digested plasmid pML523. The pHL015 harbors a kanamycin resistance gene (aph, from pIKM1), a Bacillus subtilis replicon (pIM-ori, from pIKM1), a hygromycin resistance gene (hyg), a green fluorescence protein gene (gfp), a catechol 2,3-dioxygenase gene (xylE), a levansucrase gene (sacB) and an E. coli replicon (pUC-ori). (0.24 MB TIF) [file pone.0012582.s002.tif]

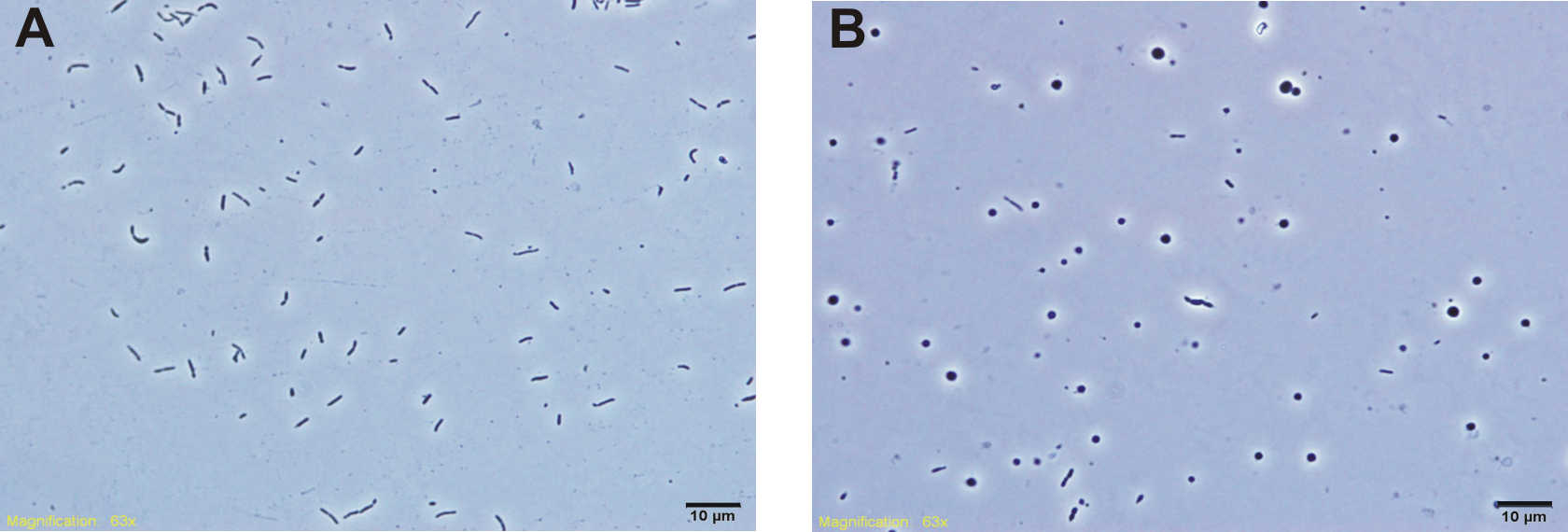

Supplement: Figure S3 — Morphology of Thermoanaerobacter sp. X514 cells. A: X514 cells at early growth phase. B: Spheroplast formation of X514 cells in the presence of glycine and sucrose. Photographs were taken using Olympus-BX51 microscope equipped with a CCD. Scale bar represents 10 µm. (1.13 MB TIF) [file pone.0012582.s003.tif]
